# Linking glucose metabolism to arterial stiffness and blood pressure in non-diabetic adults: the mediating role of FGF21

**DOI:** 10.3389/fcvm.2026.1856676

**Published:** 2026-07-14

**Authors:** Katarina Zakic, Christa Meisinger, Jakob Linseisen, Dennis Freuer

**Affiliations:** Epidemiology, Medical Faculty, University of Augsburg, Augsburg, Germany

**Keywords:** arterial stiffness, augmentation index, blood pressure, FGF21, glucose metabolism, pulse wave velocity

## Abstract

**Background and aims:**

Although glucose levels are routinely used in the diagnosis and management of diabetes, their relevance in non-diabetic individuals remains underexplored. Considering the mediating role of fibroblast growth factor 21 (FGF21), this study examined the associations between plasma markers of glucose metabolism and blood pressure as well as vascular health in a population-based sample.

**Methods and results:**

In 228 non-diabetic adults from the MEGA study, multivariable linear regression models were used to examine the associations of fasting glucose, two-hour glucose levels following an oral glucose tolerance test, and HbA1c with blood pressure (BP) parameters, pulse wave velocity, augmentation index (AIx), peripheral augmentation index (pAIx), and central aortic pressure. Effect modifications of age, sex, and BMI and the mediation mechanism of FGF21 were investigated. Two-hour glucose levels were positively associated with systolic BP [β = 2.23; 95% CI (0.29; 4.17); *P* = 0.024], mean arterial pressure [β = 1.70; 95% CI (0.07; 3.34); *P* = 0.042], diastolic BP [β = 1.48; 95% CI (0.06; 2.91); *P* = 0.041], and AIx [β = 0.96; 95% CI (0.06; 1.86); *P* = 0.037] before but not after adjusting for multiple testing. Furthermore, there was an association with systolic BP among non-obese individuals and with pAIx in participants younger than 40 years. No notable mediation effects of FGF21 were found in any of the associations.

**Conclusion:**

In this population-based sample of non-diabetic adults, higher post-challenge glucose levels showed suggestive associations with blood pressure and vascular function, particularly in non-obese individuals and younger participants. However, these associations did not remain statistically significant after correction for multiple testing, indicating that the findings should be interpreted cautiously.

## Introduction

Disturbances in glucose metabolism are closely linked to increased risk of cardiovascular disease and impaired vascular function (1). Chronic hyperglycemia and insulin resistance are known to promote endothelial dysfunction, increase arterial stiffness, and contribute to the development of elevated blood pressure (2). Early vascular ageing, characterized by reduced arterial elasticity, is frequently observed in individuals with hypertension and in those with a higher burden of cardiovascular risk factors (3, 4). One of the key manifestations of vascular ageing is arterial stiffening, which can be assessed by increased pulse wave velocity (PWV), augmentation index (AIx), and central aortic pressure (5–7). In addition, elevated blood pressure itself represents an important risk factor for cardiovascular complications in individuals with metabolic disturbances (8). Prior research mostly conducted in patients with prediabetes or diabetes has shown that PWV and blood pressure are consistently influenced by glucose metabolism, while evidence regarding AIx is heterogeneous (9–12). However, population-based studies including non-diabetic subjects are scarce and inconsistent, and there is limited research examining the associations between individual glucose parameters and vascular parameters, which could help identify the most reliable early marker of arterial stiffness (10, 13–16).

In recent years, increasing attention has been directed toward metabolic hormones that may provide a mechanistic link between metabolic disorders and cardiovascular dysfunction (17). Fibroblast Growth Factor 21 (FGF21) is a circulating endocrine hormone predominantly produced by the liver and plays an important role in the regulation of glucose and lipid metabolism, insulin sensitivity, and overall energy homeostasis (18). Elevated circulating concentrations of FGF21 have been reported in several cardiometabolic conditions, including type 2 diabetes, metabolic syndrome, non-alcoholic fatty liver disease and coronary artery disease (19, 20).

Apart from this, FGF21 may influence vascular function and blood pressure regulation through multiple distinct mechanisms (21). By improving insulin sensitivity and lipid metabolism, FGF21 reduces metabolic stress, oxidative damage, and chronic inflammation (22). The evidence supports that its anti-inflammatory actions and endothelial protective effects enhance nitric oxide bioavailability and endothelial-dependent vasodilation (23). These changes improve vascular function, reduce peripheral vascular resistance, and ultimately contribute to the regulation of blood pressure (23–25). However, the extent to which FGF21 explains the relationship between disturbances in glucose metabolism and vascular parameters such as arterial stiffness and blood pressure remains unclear (26).

Therefore, the aim of this study was to investigate the associations between markers of glucose metabolism [fasting glucose, two-hour oral glucose tolerance test (OGTT) glucose, HbA1c] and vascular parameters, including various blood pressure parameters as well as indices indicative of arterial stiffness [PWV, AIx, peripheral AIx (pAIx), central aortic pressure] in a non-diabetic population-based sample. In addition, we sought to explore the potential mediating role of FGF21 in these associations.

## Methods

The study was based on data from the MEGA study (German acronym for metabolic health study Augsburg), a population-based study conducted in Augsburg, Germany. A total of 238 men and women aged 25–65 years were interviewed and examined up to four points within a period of 9 months (baseline visit, follow-up visits after 1 and 6 months and a final visit after 9 months) between 2018 and 2021. The present analysis used data from 228 non-diabetic participants at the baseline visit, during which comprehensive phenotyping of all study participants was carried out. In addition to standardized questionnaires (e.g., collecting information on lifestyle, sociodemographic data, and previous illnesses), all participants underwent physical examinations. In addition, laboratory data, results of oral glucose tolerance tests, and long-term glucose measurements were available. Detailed information regarding the MEGA study can be found in a previous publication (27). All examinations were performed in an at least 12-h overnight fasting state and carried out by trained study nurses in accordance with previously defined standard operating procedures.

All study participants gave written informed consent. Methods of data and bio-sample collection have been approved by the ethics committee of the Ludwig-Maximilians Universität München and the study was performed in accordance with the Declaration of Helsinki. The study was registered at the DRKS (“Deutsches Register Klinischer Studien”) with the project number DRKS00015784.

### Exposures

The following glucose measurements were determined as exposures: fasting plasma glucose levels, two-hour plasma glucose after ingestion of 75 g glucose in the framework of an oral glucose tolerance test in non-diabetic participants, and the HbA1c value. For the blood collection, NaF/citrate plasma tubes (GlucoEXACT) were used, and the measurements were performed at the laboratory of the University Hospital Augsburg immediately after collection. HbA1c was measured by a reverse-phase cation-exchange high-pressure liquid chromatography (HPLC, Analyzer HA 8160; Menarini, Florence, Italy).

### Outcomes

The Vicorder device (SMT medical technology GmbH & Co. KG, Würzburg) was used to measure and characterize arterial stiffness. The PWV defined as the speed of the pulse wave between the Arteria carotis communis in the neck and the A. femoralis in the thigh was determined after the participant was resting in supine position for at least 10 min. Furthermore, the AIx, which quantifies the contribution of wave reflections to central systolic and pulse pressure, and the pAIx, reflecting the degree of pulse wave reflection in peripheral arteries, were measured. Furthermore, central aortic blood pressure (AOBP) was determined; this measurement reflects the pressure in the central aorta, which differs from peripheral (brachial) blood pressure and more accurately represents the pressure experienced by vital organs such as the heart, brain, and kidneys (28). Blood pressure was measured after a 5-minute rest period. A total of three blood pressure measurements were taken at 2-minute intervals, and for analysis, the average of the second and third measurements of systolic (SBP) and diastolic blood pressure (DBP) was used. Additionally, pulse pressure (PP), defined as the difference between systolic and diastolic arterial blood pressure, and mean aortic pressure (MAP), representing the average arterial pressure throughout one cardiac cycle, were measured (29). All outcomes were obtained from all study participants, irrespective of hypertension or antihypertensive medication use.

### Measurement of FGF21

FGF21 levels were quantified as one of 92 protein markers analyzed in EDTA plasma using the Olink inflammation panel. This analysis relies on the Proximity Extension Assay (PEA) technology. Further details about the methodology are available on the Olink Proteomics website and in a previously published study (30).

### Statistical analysis

Depending on their distributions, continuous variables were presented either as mean and standard deviation (SD) or as median and interquartile range (IQR). Normally distributed variables were analyzed using t-tests, otherwise Mann–Whitney-U tests were applied. Categorical variables were presented as absolute frequencies and column-wise percentages and analyzed using $\chi^{2}$-tests.

Multivariable linear regression models were chosen to examine the associations of fasting glucose, 2-h glucose during an OGTT, and HbA1c levels (exposures) with AOBP, SBP, DBP, MAP, PP, AIx, pAIx, PWV (outcomes). To ensure comparability of the effect sizes, the exposure variables were standardized (i.e., $\frac{X-\mu }{\sigma }$, so that X∼N(0,1)). All the assumptions of linear regression were checked and ensured. Normal distributions of the regression-specific residuals were checked graphically using both histograms and Q-Q plots. The linearity assumption between each continuous covariate and the respective outcome was assessed using polynomials of degree 2 (i.e., adding and testing the squared terms). Potential outliers were investigated and identified using Cook´s distance.

Based on literature review, potential confounding factors were selected, and their relationships were modeled using a directed acyclic graph (DAG; Supplementary Figure S1). Therefore, all models were adjusted for the following potential confounding variables: age (years), body mass index (BMI, kg/m²), sex (women, men), smoking status (current smoker, former smoker, never smoker), risky alcohol consumption (defined as AUDIT-C-Score ≥ 3 for women or ≥ 4 for men), physical activity (low, moderate, high) and education (low/medium, high).

**Figure 1:**
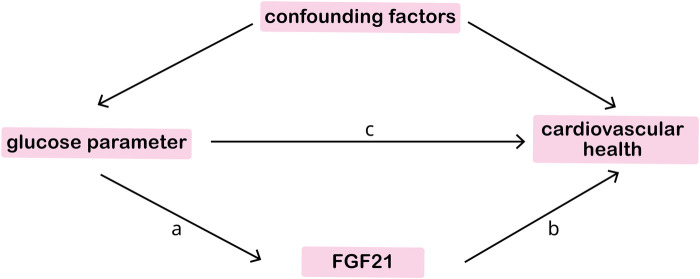
Simplified directed acyclic graph (DAG) showing the setting of mediation analyses with FGF21 served as mediator for the associations between glucose parameters (exposure) and cardiovascular health (outcome). Total effect is represented by all paths **(a,b,c)** from exposure to outcome. Path c represents the direct effect of exposure on outcome. Path a*b represents the indirect effect from exposure to outcome through FGF21. The complete DAG can be found in Supplementary Figure S1.

Effect modification of age, BMI, and sex was assessed by including and testing the respective multiplicative interaction terms with the exposure in each regression model based on α = 0.05. When there was an indication of effect modification, marginal effect estimates were calculated. Mediation analyses were conducted to examine whether the associations between glucose parameters and the outcomes were mediated by FGF21 (Figure 1). Direct and indirect effects were estimated and tested based on α = 0.05. Where appropriate, the proportion mediated was calculated. The Huber-White method was applied to calculate heteroscedasticity-robust standard errors for the particular paths and the confidence intervals for the indirect effects were estimated using 5,000 bootstrap samples.

The reported effect estimates [β-coefficient and 95% confidence interval (CI)] represent the expected change in an outcome associated with one SD increase in the exposure variable. Since there were only occasional missing values and their mechanism can be considered completely at random, a complete case analysis was performed.

In terms of the number of exposures and outcomes, there were a total of 24 hypotheses to be tested. Therefore, *P* values in the main analyses were adjusted for multiple testing using the FDR (false discovery rate) approach (denoted as P_FDR_) and results were evaluated based on the significance threshold of α = 0.05. All analyses were performed using R (version 4.5.1).

## Results

### Descriptive analyses

Table 1 presents the characteristics of both the total sample and stratified by AIx. The median age of study participants was 47 years, and their median BMI was 25 kg/m². Less than half of the participants reported low to moderate levels of physical activity, and about two-thirds had a low to medium level of education. Participants with higher AIx were older, had higher BMI and were more prone to hypertension. In addition, they had higher total and LDL cholesterol levels and higher values in all outcome variables.

**Table 1:** Baseline characteristics of total sample and stratified by augmentation index.

Characteristic	Total sample (*n* = 228)	AIx < 20 (*n* = 100)	AIx ≥ 20 (*n* = 128)	P
Age (years)	47 (35, 55.5)	36 (29, 45.75)	53 (47, 59)	< 0.001
BMI (kg/m²)	25 (23, 33)	25 (22, 31)	28 (23, 34)	0.036
PWV (m/s)	7.54 ± 1.35	6.96 ± 1.12	8.10 ± 1.34	< 0.001
AOBP (mmHg)	130 (119, 142)	121 (112, 135)	137 (126, 148)	< 0.001
SBP (mmHg)	116 (107, 128)	114 (105, 127)	119 (109, 129)	0.032
DBP (mmHg)	78 (71, 86)	76 (69, 82.75)	80 (72, 87)	< 0.001
MAP (mmHg)	93 (86, 103)	87 (81, 100)	98 (90, 105)	< 0.001
PAIx	94 (90, 97)	89.5 (87, 92)	97 (95, 98)	< 0.001
PP (mmHg)	61 (53, 72)	57 (51, 64)	66 (57, 77)	< 0.001
Fasting glucose (mg/dL)	97 (92, 103)	95 (89, 101)	99 (93, 107)	< 0.001
2-h glucose (mg/dL)	105 (87, 127)	100 (82, 119)	108 (89, 135)	0.024
HbA1c (mmol/mol)	35 (32, 37)	34 (31, 36)	35 (33, 38)	< 0.001
Total cholesterol (mg/dL)	186.8 ± 35.4	174.4 ± 33.2	197.5 ± 34.0	< 0.001
LDL cholesterol (mg/dL)	120.3 ± 31.3	109.8 ± 30.4	128.8 ± 29.4	< 0.001
HDL cholesterol (mg/dL)	60 (49, 73)	61 (49, 74)	60 (49, 73)	0.248
Triglycerides (mg/dL)	81 (59, 122)	73 (53, 124)	92 (70, 123)	0.119
Hypertension				0.007
No	172 (75.1%)	85 (85.0%)	89 (76.9%)	
Yes	56 (24.9%)	15 (15.0%)	39 (33.1%)	
Smoking				0.434
Current	32 (14.2%)	14 (14.0%)	16 (13.6%)	
Former	79 (35.1%)	30 (30.0%)	49 (41.5%)	
Never	114 (50.7%)	56 (56.0%)	57 (44.9%)	
Risky alcohol use^a^				0.062
No	117 (50.7%)	55 (55.0%)	75 (55.1%)	
Yes	111 (49.3%)	45 (45.0%)	53 (44.9%)	
Physical activity				0.152
Low	32 (14.2%)	12 (12.0%)	19 (16.1%)	
Moderate	65 (28.9%)	26 (26.0%)	36 (30.5%)	
High	127 (56.4%)	62 (62.0%)	62 (52.5%)	
Education				0.153
Low/Medium	158 (70.2%)	62 (62.0%)	90 (76.3%)	
High	67 (29.8%)	38 (38.0%)	28 (23.7%)	

Continuous variables were presented as mean (± SD) or median (25th, 75th percentiles). Categorical variables were given as *n* (%).

AIx, augmentation index; AOBP, aortic blood pressure; BMI, body mass index; DBP, diastolic blood pressure; MAP, mean arterial pressure; pAIx, peripheral augmentation index; PP, pulse pressure; PWV, pulse wave velocity; SBP, systolic blood pressure.

a Defined as AUDIT-C-Score ≥ 3 for women or ≥ 4 for men.

### Association analyses

**Figure 2:**
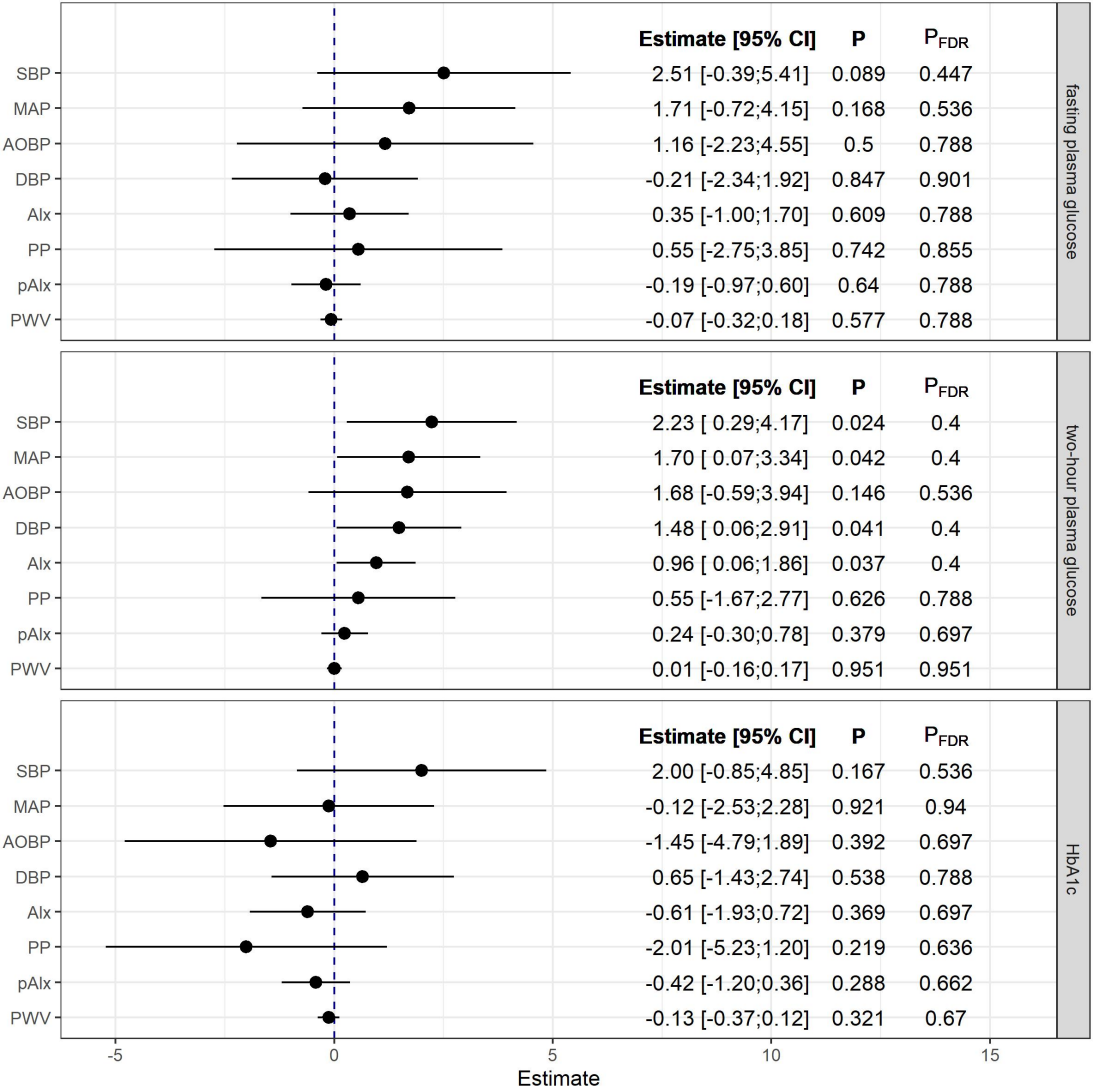
Associations between plasma markers of glucose metabolism and parameters indicative of cardiovascular changes in non-diabetic participants estimates and 95% confidence intervals (CIs) were obtained from linear models adjusted for age, sex, body mass index, smoking status, alcohol consumption, physical activity and education. Point estimates can be interpreted as the mean change in the respective outcome per one standard deviation increase in the exposure. Both raw and FDR-adjusted *P* values were presented. AIx, augmentation index; AOBP, aortic blood pressure; DBP, diastolic blood pressure; FDR, false discovery rate; MAP, mean aortic pressure; SBP, systolic blood pressure; pAIx, peripheral augmentation index; PP, pulse pressure; PWV, pulse wave velocity.

Figure 2 presents the results from multivariable regression analyses. Two-hour plasma glucose levels were associated with SBP [β = 2.23; 95% CI (0.29; 4.17); *P* = 0.024], MAP [β = 1.70; 95% CI (0.07; 3.34); *P* = 0.042], DBP [β = 1.48; 95% CI (0.06; 2.91); *P* = 0.041], and AIx [β = 0.96; 95% CI (0.06; 1.86); *P* = 0.037]. However, after adjusting for multiple testing, the initial associations failed to reach statistical significance. No associations in form of total effects were found with the remaining outcomes.

### Effect modification analyses

**Figure 3:**
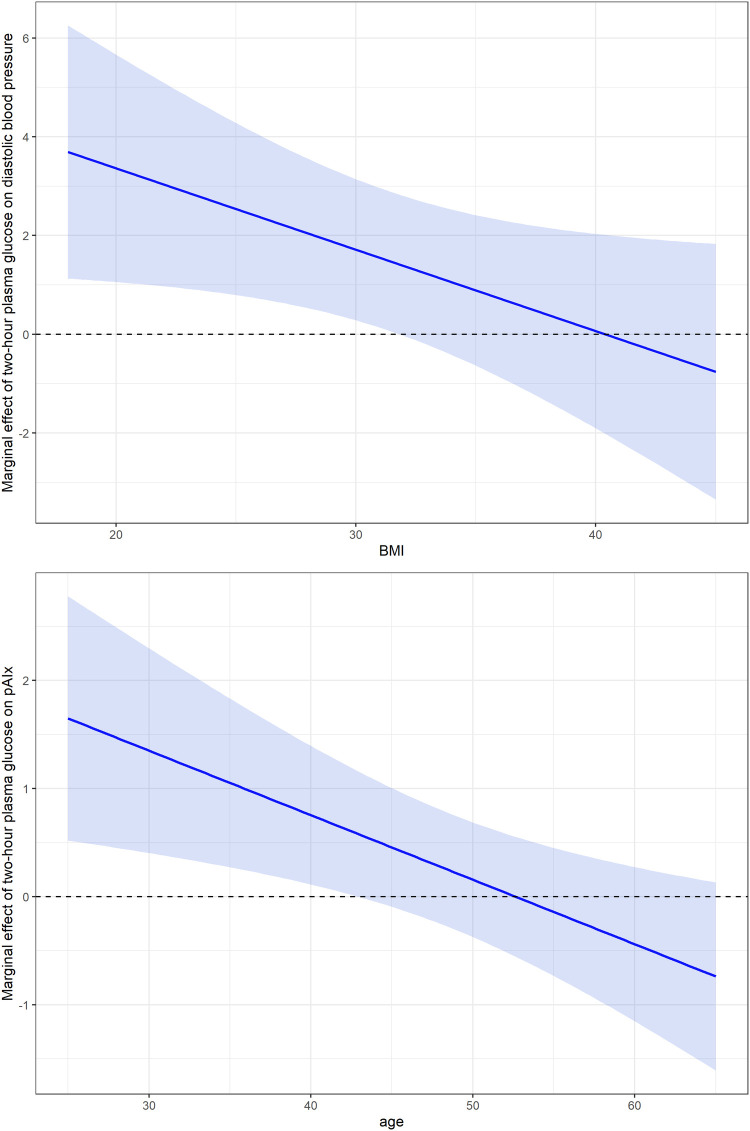
Marginal effect estimates of the relationship between two-hour glucose levels and both diastolic blood pressure and peripheral augmentation index (pAIx). Estimates and 95% confidence bands [based on body mass index (BMI) or age] were obtained from linear models adjusted for age, sex, BMI, smoking status, alcohol consumption, physical activity, education and the respective interaction term (between exposure and BMI or age). Point estimates (represented by blue lines) can be interpreted, depending on the BMI or age, as the mean change in the respective outcome per one standard deviation increase in the exposure. The dashed horizontal line indicates the null hypothesis of no association.

Based on evidence from interaction tests, the subsequent analyses revealed a BMI-specific difference in the relationship of two-hour plasma glucose with DBP (P_interaction_ = 0.043), where a positive association was found primarily among non-obese individuals (Figure 3). As indicated by the marginal effects, the strength of this association decreased continuously with increasing BMI. A similar pattern was found for the association between two-hour glucose and pAIx (P_interaction_ = 0.006) among participants under approximately 40 years of age (Figure 3). There was no evidence of sex-dependent differences in the relationships examined.

### Mediation analyses

Despite a notable association between two-hour plasma glucose levels and FGF21 [β = 0.21; 95% CI (0.04; 0.37); *P* = 0.016], the *a priori* hypothesized mediating effect could not be confirmed for any of the associations described above (Supplementary Table S1). This was mainly due to the lack of associations between FGF21 and the respective outcomes.

## Discussion

In this population-based study, two-hour plasma glucose levels were positively associated with SBP, MAP, DBP, and AIx; however, these associations did not remain statistically significant after adjustment for multiple testing. Notably, BMI- and age-specific differences emerged: a positive association between two-hour glucose and DBP was found among non-obese individuals, and an association between two-hour glucose and pAIx was observed in participants younger than 40 years. FGF21 did not mediate any of these associations.

Previous studies reported a positive association between diabetes and elevated AIx (12, 31, 32), while others have found no relationship between HbA1c levels and AIx (12). However, so far studies addressing AIx and pAIx in non-diabetic individuals are scarce. One available study by Schneider et al. demonstrated that the 2-h OGTT glucose value was superior to fasting glucose in predicting central augmentation index (cAIx) in young high-risk individuals, a finding which is supported by the present results regarding the point estimates (33). Furthermore, the results of our stratified analyses indicate an age-dependent association between two-hour OGTT glucose levels and pAIx. A previous study showed that AIx increased non-linearly with age, suggesting age-dependent patterns in AIx (34). However, so far, no study has examined whether the glucose-AIx association varies by age-group.

The AIx is a measure of systemic arterial stiffness derived from the ascending aortic pressure waveform (35). Several studies have found that increased arterial stiffness may predict future cardiovascular morbidity and mortality in individuals with diabetes (36, 37). However, some prior studies have reported contrary results, with radial augmentation index paradoxically being lower in patients with diabetes despite the presence of advanced atherosclerotic changes (38). This apparent contradiction may be explained by preferential stiffening of large (central) arteries, which reduces impedance mismatch between central and peripheral arteries and thereby diminishes the magnitude of wave reflection (39).

Although these mechanisms were primarily described in diabetes, our findings indicate that vascular alterations related to glucose metabolism could be present in non-diabetic individuals. Despite the lack of statistical significance after adjusting for multiple testing in the present study, two-hour OGTT glucose showed initial associations with certain vascular parameters, extending beyond fasting glucose measures. This is consistent with previous evidence indicating that post-challenge glucose levels are more strongly associated with cardiovascular complications than fasting glucose. For example, Kodama et al. found that post-challenge glucose was approximately 50% more strongly associated with cardiovascular disease risk compared to fasting glucose (40). Another study showed that post-challenge hyperglycemia was associated with arterial stiffness in non-diabetic men and women (41).

However, the specific mechanisms linking post-challenge glucose to AIx remain incompletely understood. It seems possible that post-challenge glucose excursions may cause more acute vascular damage (42, 43), and thus reflect metabolic variability more comprehensively than static fasting glucose measurement (44), or even indicate underlying insulin resistance and metabolic dysfunction (45, 46).

Previous studies showed a strong positive association between diabetes and hypertension (47, 48), with the risk being higher in those with impaired fasting glucose rather than in those with abnormal HbA1c levels (49). In the present population-based study, no associations between HbA1c or fasting plasma glucose levels and various blood pressure measures were obtained. We observed only an initial association (loosing significance after adjusting for multiple testing) between two-hour OGTT glucose and DBP. In prior studies two-hour OGTT glucose was associated with hypertension development (50, 51) and blood pressure (52), with a stronger relationship to systolic than diastolic blood pressure, which are generally consistent with our findings. On the other hand, contrary to our findings regarding the BMI-dependent association between two-hour OGTT glucose and DBP, a previous study found stronger associations in men with higher BMI (53). Regarding the loss of statistical significance after adjustment for multiple testing, the observed associations should be interpreted cautiously and requires confirmation in further studies.

Some population-based studies reported associations between blood glucose parameters and PWV. For instance, Webb et al. found that both fasting glucose (β = 0.10) and two-hour post-challenge glucose (β = 0.14) were independently related to carotid-femoral PWV (10). Koivistoinen et al. further confirmed that two-hour glucose levels during an OGTT were an independent determinant of PWV (*P* = 0.005) in a population-based but not strictly non-diabetic sample (54). In contrast, we did not observe an association between any of the three glucose parameters and PWV.

In the present study, PWV was measured non-invasively using the oscillometric Vicorder system. Alternatively, estimated pulse wave velocity (ePWV), which is derived from age and mean arterial pressure, provides a practical surrogate measure of arterial stiffness for epidemiological studies. Previous research has demonstrated that ePWV is associated with all-cause mortality as well as with measured PWV in young and middle-aged adults with moderate cardiopulmonary health status (55). Also, ePWV was independently associated with all-cause and cardiovascular mortality in large population-based cohorts (56, 57). In contrast, measured PWV directly quantifies arterial properties, captures interindividual differences in vascular aging that are not explained by age and blood pressure alone, and is therefore better suited for mechanistic research (58, 59). Consequently, for investigating the relationship between glucose metabolism and arterial stiffness, as well as the potential mediation role of FGF21, measured PWV remained the preferred approach in the present study.

In this study, HbA1c levels within the non-diabetic range were not associated with any of the vascular outcome variables. This is consistent with some previous studies conducted in patients with prediabetes or diabetes (12, 49). In contrast, a study in non-diabetic Chinese adults found that HbA1c showed a stronger association with PWV than fasting glucose and 2-h OGTT glucose (60). While HbA1c is a useful tool for diabetes management, it has several notable limitations (60). It is a relatively crude measure of glycemic control, as a single HbA1c value may reflect a wide range of glucose levels and does not capture extreme glucose values or variability. Additionally, HbA1c levels can be falsely elevated or reduced due to various medical conditions and substance use (61).

FGF21 did not mediate any of the associations investigated in this study, because it was not associated with the respective outcomes. Contrary, a cross-sectional study of 744 community-dwelling adults found serum FGF21 independently associated with hypertension (62). In another study, FGF21 serum levels were significantly associated with systolic blood pressure and fasting plasma glucose levels, suggesting a potential link between FGF21 and both glucose metabolism and blood pressure regulation (63). Furthermore, circulating FGF21 levels have been reported to increase in individuals with impaired glucose metabolism and metabolic dysfunction (64). In addition, experimental studies also suggested that FGF21 may influence cardiovascular regulation through metabolic and vascular pathways (65, 66). Further prospective studies are needed to clarify the role of FGF21 in the link between glucose metabolism and vascular health.

A strength of this study is that it expands existing knowledge on the relationship between glucose parameters and vascular health by analyzing associations between specific glycemic parameters and vascular parameters in non-diabetic subjects. This approach may help identify the most reliable early marker of arterial stiffness. Additionally, the study included a relatively large number of well-phenotyped individuals, and all measurements were highly standardized. The comprehensive data collection for each participant allowed for adjustment for relevant confounders. However, as the majority of the study sample consisted of individuals from a typical German population aged 25–65 years, the results may not be generalizable to other ethnicities or age groups. Another limitation is the cross-sectional design, which precludes establishing causality and raises the possibility of reverse causation, although the direction of effect is generally understood. In addition, the inclusion of people with known hypertension and those with known antihypertensive medication could have biased the results.

## Conclusions

In conclusion, this population-based study suggests that higher two-hour OGTT glucose levels may be associated with adverse blood pressure profiles and increased wave reflection, particularly in non-obese individuals and younger participants. However, these associations did not remain statistically significant after correction for multiple testing, indicating that the findings should be interpreted cautiously. No evidence was found for the mediating role of FGF21 in these relationships. Overall, the results point toward potential early vascular and hemodynamic alterations related to post-challenge glycemia in selected subgroups, but require confirmation in larger and longitudinal studies.

## Data Availability

The raw data supporting the conclusions of this article will be made available by the authors, without undue reservation.
