# Supplementary material for: Linking glucose metabolism to arterial stiffness and blood pressure in non-diabetic adults: the mediating role of FGF21
Source: Front Cardiovasc Med. 2026 Jul 14;13:1856676. doi: 10.3389/fcvm.2026.1856676 (PMC13407631; doi:10.3389/fcvm.2026.1856676)
Supplement: Supplementary file 1 [file Supplementaryfile1.docx]

| **Supplementary Table 1** Results of mediation analyses assessing the mediation effects of FGF21 on the association between two-hour plasma glucose levels and blood pressure as well as vascular health. Analyses were performed for total effect estimates with P < 0.05 arising from multivariable linear regression models. | | | | | | | | | | | |
| --- | --- | --- | --- | --- | --- | --- | --- | --- | --- | --- | --- |
|  |  |  | **Total Effect (X->Y)** | | | |  | **Direct Effect (X->Y)** | | | |
| **Exposure** | **Outcome** |  | **Beta** | **cil** | **ciu** | **P** |  | **Beta** | **cil** | **ciu** | **P** |
| OGTT | SBP |  | 2.23 | 0.29 | 4.17 | 0.024 |  | 1.95 | -0.03 | 3.93 | 0.053 |
| OGTT | MAP |  | 1.70 | 0.07 | 3.34 | 0.042 |  | 1.75 | 0.07 | 3.42 | 0.041 |
| OGTT | DBP |  | 1.48 | 0.06 | 2.91 | 0.041 |  | 1.27 | -0.18 | 2.73 | 0.085 |
| OGTT | AIx |  | 0.96 | 0.06 | 1.86 | 0.037 |  | 0.89 | -0.04 | 1.82 | 0.059 |
|  |  |  | **X->M** | | | |  | **M->Y** | | | |
| **Exposure** | **Outcome** |  | **Beta** | **cil** | **ciu** | **P** |  | **Beta** | **cil** | **ciu** | **P** |
| OGTT | SBP |  | 0.21 | 0.04 | 0.37 | 0.016 |  | 1.06 | -0.62 | 2.73 | 0.215 |
| OGTT | MAP |  | 0.21 | 0.04 | 0.37 | 0.016 |  | -0.49 | -1.91 | 0.93 | 0.499 |
| OGTT | DBP |  | 0.21 | 0.04 | 0.37 | 0.016 |  | 0.66 | -0.57 | 1.88 | 0.291 |
| OGTT | AIx |  | 0.21 | 0.04 | 0.37 | 0.016 |  | 0.24 | -0.55 | 1.03 | 0.543 |


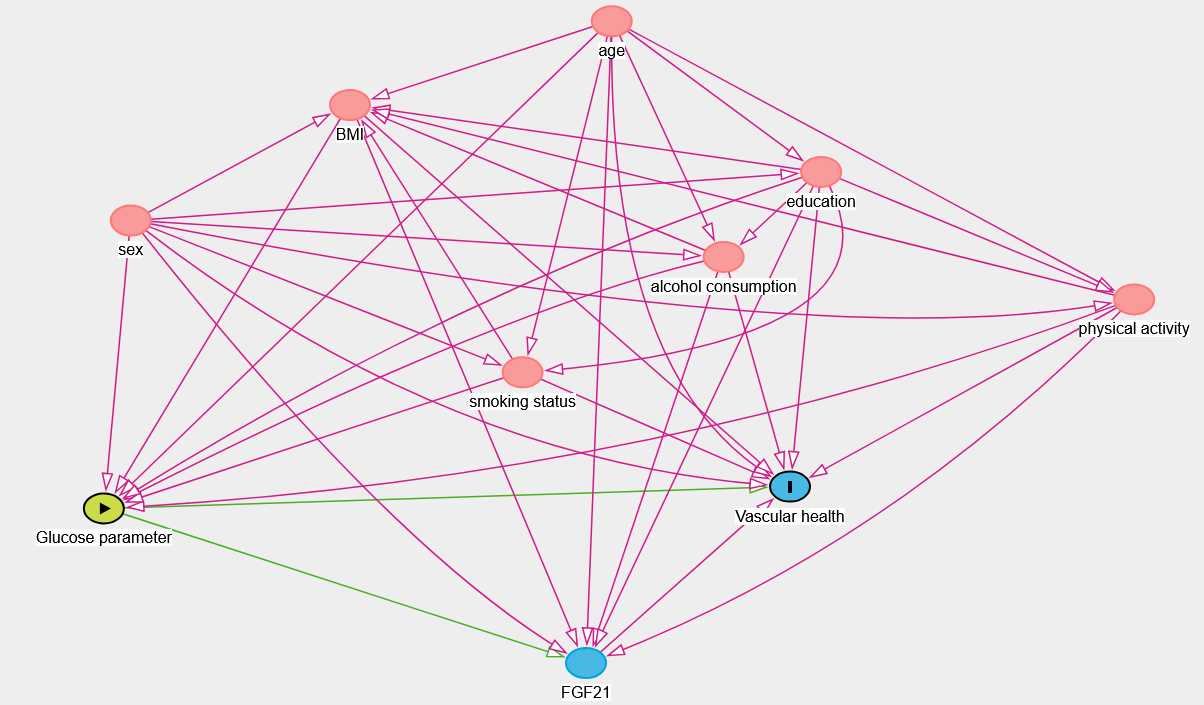


**Supplementary Figure 1** Directed acyclic graph of the associations between glucose metabolism and arterial stiffness as well as blood pressure in non-diabetics.
